# The Combination of Hemogram Indexes to Predict Exacerbation in Stable Chronic Obstructive Pulmonary Disease

**DOI:** 10.3389/fmed.2020.572435

**Published:** 2020-12-09

**Authors:** Xuanqi Liu, Haiyan Ge, Xiumin Feng, Jingqing Hang, Fengying Zhang, Xiaoyan Jin, Hong Bao, Min Zhou, Fengfeng Han, Shengqing Li, Yechang Qian, Zhijun Jie, Wenchao Gu, Beilan Gao, Li Yu, Jian Wang, Haiying Ji, Jingxi Zhang, Huili Zhu

**Affiliations:** ^1^Department of Respiratory and Critical Care Medicine, Huadong Hospital, Fudan University, Shanghai, China; ^2^Department of Respiratory and Critical Care Medicine, Changji Branch of First Affiliated Hospital of Xinjiang Medical University, Xinjiang, China; ^3^Department of Respiratory Medicine, Putuo People's Hospital, Shanghai, China; ^4^Department of Respiratory Medicine, Tongren Hospital, Shanghai Jiaotong University School of Medicine, Shanghai, China; ^5^Department of Respiratory Medicine Shanghai Pudong Hospital, Fudan University Pudong Medical Center, Shanghai, China; ^6^Department of Respiratory and Critical Care Medicine, Ruijin Hospital, Shanghai Jiaotong University School of Medicine, Shanghai, China; ^7^Department of Respiratory Medicine, Xinhua Hospital, Shanghai Jiaotong University School of Medicine, Shanghai, China; ^8^Department of Respiratory and Critical Care Medicine, Huashan Hospital, Fudan University, Shanghai, China; ^9^Baoshan District Hospital of Integrated Traditional Chinese and Western Medicine Hospital, Shanghai, China; ^10^Department of Respiratory Medicine, Shanghai Fifth's Hospital, Fudan University, Shanghai, China; ^11^Department of Respiratory Medicine, Pudong New District People's Hospital, Shanghai, China; ^12^Department of Respiratory Medicine, Shanghai Pulmonary Hospital, Tongji University, Shanghai, China; ^13^Department of Pulmonary and Critical Care Medicine, Tongji Hospital, Tongji University School of Medicine, Shanghai, China; ^14^Department of Respiratory Medicine, Shanghai Ninth's Hospital, Shanghai Jiaotong University School of Medicine, Shanghai, China; ^15^Department of Respiratory and Critical Care Medicine, Zhongshan Hospital, Fudan University, Shanghai, China; ^16^Department of Respiratory and Critical Care Medicine, Changhai Hospital Affiliated to Navy Military Medical University, Shanghai, China

**Keywords:** COPD, hemogram index, PLR, exacerbation, inflammation

## Abstract

**Background:** Chronic obstructive pulmonary disease (COPD) is characterized by pulmonary and systemic inflammatory processes, and exacerbation of COPD represents a critical moment in the progression of COPD. Several biomarkers of inflammation have been proposed to have a predictive function in acute exacerbation. However, their use is still limited in routine clinical practice. The purpose of our study is to explore the prognostic efficacy of novel inflammatory hemogram indexes in the exacerbation among stable COPD patients.

**Method:** A total of 275 stable COPD patients from the Shanghai COPD Investigation Comorbidity Program were analyzed in our study. Blood examinations, especially ratio indexes like platelet–lymphocyte ratio (PLR), platelet × neutrophil/lymphocyte ratio [systemic immune-inflammation index (SII)], and monocyte × neutrophil/lymphocyte ratio [systemic inflammation response index (SIRI)], lung function test, CT scans, and questionnaires were performed at baseline and routine follow-ups. Clinical characteristics and information of exacerbations were collected every 6 months. The relationship between hemogram indexes and diverse degrees of exacerbation was assessed by logistic regression. The receiver operating characteristic (ROC) curve and area under the curve (AUC) were used to evaluate the ability of hemogram indexes to predict exacerbation of COPD. Furthermore, the discrimination and accuracy of combined indexes were measured by ROC and calibration curve.

**Result:** There was a significant positive correlation between PLR levels and total exacerbation of COPD patients in a stable stage in a year. Also, the predictive ability of PLR exceeded any other ratio indexes, with an AUC of 0.66. SII and SIRI ranked second only to PLR, with an AUC of 0.64. When combining PLR with other indexes (sex, COPD year, and St. George's Respiratory Questionnaire scores), they were considered as the most suitable panel of index to predict total exacerbation. Based on the result of the ROC curve and calibration curve, the combination shows optimal discrimination and accuracy to predict exacerbation events in COPD patients.

**Conclusion:** The hemogram indexes PLR, SII, and SIRI were associated with COPD exacerbation. Moreover, the prediction capacity of exacerbation was significantly elevated after combining inflammatory hemogram index PLR with other indexes, which will make it a promisingly simple and effective marker to predict exacerbation in patients with stable COPD.

## Introduction

Chronic obstructive pulmonary disease (COPD) is characterized by persistent airflow limitation, which is usually progressive. Chronic airway and lung inflammation do exist in COPD patients (1). It is well-known that cigarette smoke inducing chronic inflammation results in the remodeling and narrowing of small airways and the destruction of lung parenchyma in COPD. According to the World Health Organization (WHO), COPD will be the third leading cause of death in the world. In the recent few years, the exacerbation of COPD has become the focus of our attention. It contributed to a series of problems (2, 3)—worsening lung function, decreasing life quality (4, 5), accelerating disease progress, and elevating the risk of hospitalization or mortality (3, 6, 7). Recently, the use of hemogram indexes indicating systemic inflammation has been determined in clinical practice for predicting hospitalization and mortality in COPD. However, there still exist obverse and reverse judgments on the usage of hemogram indexes. It is due to the exact thresholds of hemogram indexes are totally different because the target population is derived from different study designs. Meanwhile, limited data have been presented in evaluating hemogram indexes as biomarkers in patients with stable COPD. Also, the relationship between the change in these indexes and the severity of exacerbation is still uncertain.

Beyond some genetic factors, a considerable part of exacerbation cannot be explained by infection (8, 9); infection is still regarded as one of the main reasons for exacerbation in COPD (6, 10). Therefore, ratio indexes in peripheral blood, such as platelet–lymphocyte ratio (PLR), platelet × neutrophil/lymphocyte [systemic immune-inflammation index (SII)], and monocyte × neutrophil/lymphocyte [systemic inflammation response index (SIRI)], which generally served as markers of inflammation level, are studied widely, as well as hemogram indexes like platelet counts/mean platelet volume (PLT/MPV) and platelet counts/red cell distribution width (PLT/RDW) ratios on behalf of platelet and red cell activation (11, 12). What is more important is that when combining the several indexes, they are proved to be more valuable predictors in many diseases. Systemic inflammation makers SII and SIRI have been determined to play an important role in various solid tumor like lung cancer and cardiovascular diseases (13–15). It has been established that high PLR is associated with 90-day mortality in acute exacerbations of COPD (16). By contrast, conventional inflammatory indicators like C-reactive protein and procalcitonin in peripheral blood showed low specificity in predicting exacerbation of COPD (17, 18). Also, the counting of inflammatory cells in the airway is not easy and convenient to detect. Overall, the inflammatory indicators discussed earlier in the hemogram index are easier to obtain and more cost effective. It is very meaningful to analyze the inflammation-related indicators in hemogram in COPD patients.

The primary purpose of our study is to investigate the prognostic role of hemogram indexes, especially inflammation-related ones in patients with stable COPD. We also sought to explore the relationship between hemogram indexes and the severity of the exacerbation. In addition, we tried to confirm a proper combination of indexes to predict exacerbation and evaluate corresponding predictive ability, including discrimination and accuracy.

## Methods

### Study Population

**Figure 1:**
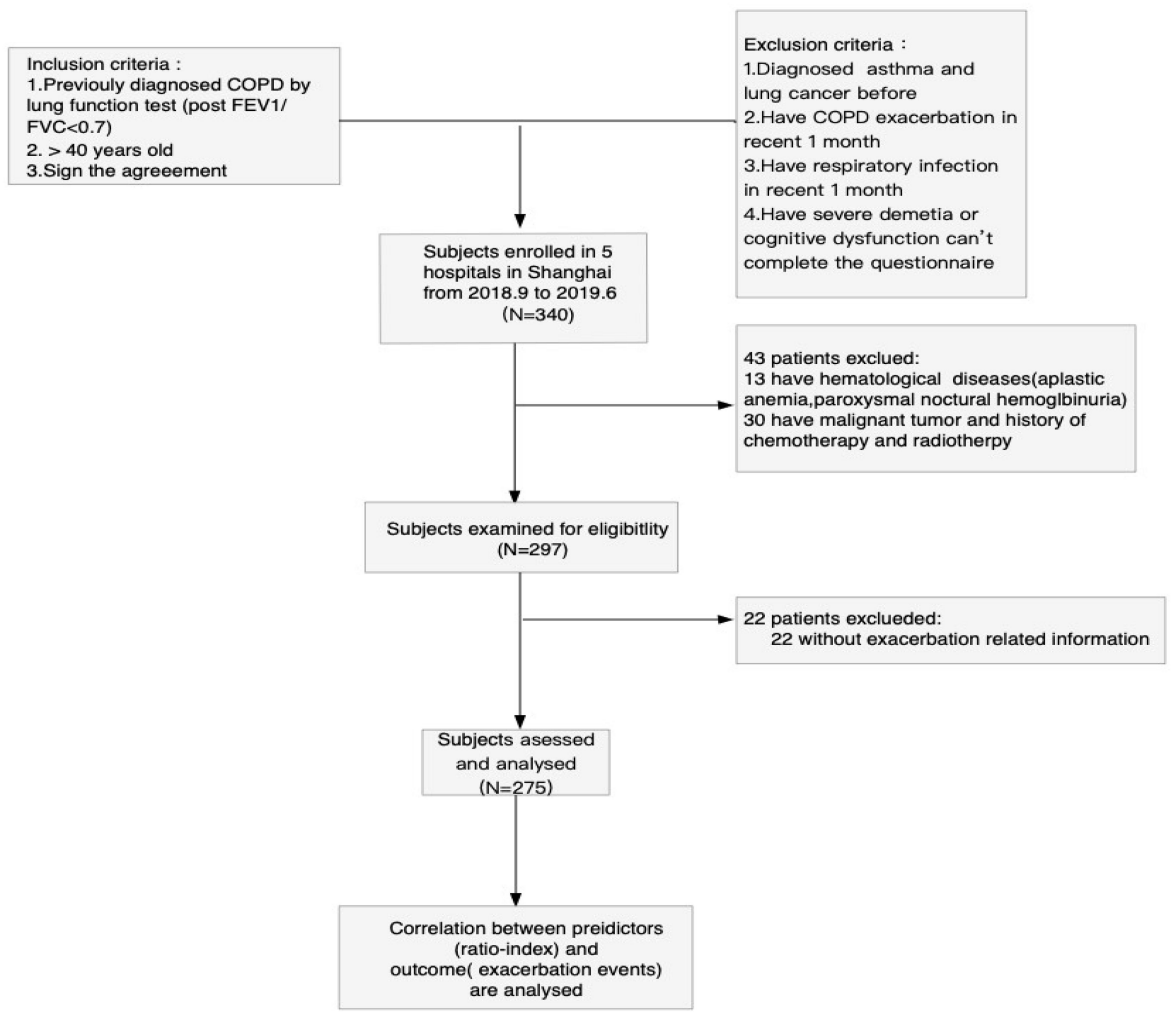
Flowchart of the study population. Process of patient selection in this study. Finally, data from 275 patients are used to analyze and assess. COPD, chronic obstructive pulmonary disease.

Patients with COPD admitted in five hospitals (Huadong Hospital, Changhai Hospital, Putuo People's Hospital, Baoshan Integrated Chinese and Western Medicine Hospital, and Pulmonary hospital) in Shanghai from June 2018 to December 2019 were enrolled. Our study has acquired the approval of the ethical committee's informed consent from Huadong Hospital. The inclusion criteria in the study were as follows: (1) Primary diagnosis of COPD in accordance with Global Initiative for Chronic Obstructive Lung Disease (GOLD) criteria (the forced expiratory volume in the first second of forced vital capacity is <70% after inhaling bronchodilators); (2) patients whose age ≥40 years; and (3) signed the agreement of participating in the study. The exclusion criteria were as follows: (1) diagnosis of asthma or lung cancer; (2) history of COPD exacerbation in recent month, which specifically refers to a deterioration of patients' respiratory tract symptoms that were beyond the normal variation and even required steroids, antibiotics, or hospitalization; (3) history of respiratory infection in the recent month; and (4) unable to complete the questionnaire due to severe dementia and cognitive dysfunction. A total of 340 stable COPD patients were initially enrolled. Then, we exclude 43 patients; among them, 13 were suffering from hematological diseases, and 30 were receiving chemotherapy or radiotherapy, which may make an influence on hemogram. At the end of the first-year follow-up, 22 patients were lost to follow-up, and their exacerbation-related information was not available. Finally, a total of 275 subjects were used to analyze and assess. The process of patient selection is shown in Figure 1.

### Study Design

Our study has been registered in the Chinese Clinical Trial Registry, and the registration number is ChiCTR2000030911. This is a 3 year prospective cohort study. The subjects in the study were required to complete blood examination, lung function tests, and questionnaires. The blood samples were collected at baseline during the stable stage. The exacerbation-related information was documented every 6 months. In our study, the definition of COPD exacerbation has been strictly identified according to GOLD 2018. The occurrence of exacerbation means acute worsening of respiratory symptoms that result in additional therapy. Mild exacerbation is treated with short-acting bronchodilators only. Moderate exacerbation should be treated with short-acting bronchodilators plus antibiotics and/or oral corticosteroids. Severe exacerbation always requires hospitalization, including emergency admission. Frequent exacerbation was defined as one who suffered from more than one exacerbation in a year. The platelet count, lymphocyte count, neutrophil count, mean platelet volume, and red cell distribution width were measured at each participating center. Then, we acquired the corresponding ratio index, such as PLR, PLT/MPV ratio, PLT/RDW ratio, SII, and SIRI. The baseline characteristics associated with the comorbidity of all the patients were recorded in the questionnaire, including COPD Assessment Test, Modified Medical Research Council, and St. George's Respiratory Questionnaire (SGRQ) scores evaluating patients' symptoms. Correlation between hemogram indexes and exacerbation events were then analyzed.

### Statistical Analysis

Data were presented as mean ± standard deviation. Logistic regression analysis was performed to evaluate the total and diverse degrees of exacerbation in stable COPD patients. To assess the index's predictive ability of exacerbation in COPD, we constructed receiver operating characteristic (ROC) curves and tabulated the area under the curves (AUC). In the section, the results of multivariate logistic regression were regarded as predictors in a total exacerbation. Logistic regression to predict exacerbation was used to determine the weight given to each standardized index. Nomograms were operated to exhibit the model's prognostic ability of total exacerbation in detail. Then, we evaluated the model's predictive capacity from two aspects. ROC curve was utilized again to test the discrimination, whereas the accuracy of the model was certified by Hosmer–Lemeshow fitting test and calibration curve. For all tests, a two-sided *P* <0.05 was considered significant. Data were analyzed by STATA 15.

## Results

### Characteristics of the Study Population

A total of 275 patients with stable COPD met our inclusion and exclusion criteria and were enrolled in this study. Table 1 provided the summary statistics for the study population. There were 243 males and 32 females, with an average age of 71 years. Of these, 22.9% were current smokers. COPD Assessment Test scores, Modified Medical Research Council scores, and SGRQ scores were conducted to assess respiratory symptoms. The patients were classified as Global Initiative for COPD (GOLD) stages 1, 2, 3, and 4. In detail, they had 33, 76, 128, and 78 cases in each group, respectively. The clinical characteristics, pulmonary function tests, and main hemogram indexes of participants were also listed in Table 1.

**Table 1:** Baseline characteristics of study patients.

**Clinical characteristic**	**COPD**
Number	275
Sex	32/243
(Female/male)	(11.6/88.4)
Age	70.8 ± 9.0
Smoking status	60/152/63
(never/ex/smoker)	(21.8/55.3/22.9)
Smoking index	33.1 ± 31.6
BMI	23.2 ± 4.1
COPD years	6.6 ± 7.7
mMRC scores	40/67/82/72/14
(1/2/3/4/5)	(14.6/24.4/29.8/26.2/5.1)
CAT score	16.6 ± 8.8
SGRQ score	35.6 ± 19.3
FEV1, %	52.2 ± 22.6
FEV1%FVC, %	56.6 ± 14.5
GOLD stage	33/76/128/78
(I/II/III/IV)	(12.0/27.6/46.6/13.8)
EOS	0.3 ± 0.9
PLR	151.1 ± 62.8
SII	745.5 ± 438.1
SIRI	1.9 ± 1.3
PLT/RDW	15.6 ± 3.9
PLT/MPV	21.5 ± 6.2

*Data were shown as mean ± SD. GOLD COPD grades are defined as: grade I: post-bronchodilator FEV1 > 80% of predicted; grade II: post-bronchodilator FEV1 50–80% of predicted; grade III: post-bronchodilator FEV1 30–50% of predicted; and grade IV: post-bronchodilator FEV1 <30% of predicted*.

*BMI, body mass index; smoking index, smoking packs per day; FEV1, forced expiratory volume in 1 s; FVC, first vital capacity; PLR, platelet–lymphocyte ratio; SII, platelet × neutrophil/lymphocyte; SIRI, monocyte × neutrophil/lymphocyte; PLT/RDW, platelet counts–red cell distribution width ratio; PLT/MPV, platelet counts–mean platelet volume ratio*.

### Relationship Between Hemogram Indexes and Exacerbation in 1 Year

Univariate analysis of logistic regression revealed that all of the hemogram indexes except PLT/RDW and PLT/MPV were associated with total exacerbation. The independence of the discussed parameters as predictors for total exacerbation was determined using the entered model of multivariate logistic regression. As a result, PLR was confirmed as an independent predictor for total exacerbation in our study (Table 2). The other parameters were not significant in multivariate regression. Next, we repeated multivariate logistic regression in different degrees of exacerbation. Interestingly, we found that the effect of PLR in severe exacerbation still existed after adjustment for known prognostic factors. SII, SIRI, and SGRQ scores also played the prognostic role in this part. The severe exacerbation seems to be more connected with hemogram indexes compared with a moderate and mild one. In addition, SII and PLT/MPV are correlated with moderate and mild exacerbation, respectively. PLT/MPV played a prognostic role in frequent exacerbation, too (Table 3). Poisson regression was performed to detect the association between hemogram indices and the number of total exacerbation in a year (Supplement 1). All of the hemogram indexes except PLR were related to the number of total acute exacerbations whether in univariate or multivariate regression. The effect of PLR maybe minimized by other mixed factors in multivariate regression.

**Table 2:** Univariate and multivariate logistic regression analysis for total exacerbation.

	**Univariate model**		**Multivariate model**	
	**OR (95% CI)**	***P*-value**	**OR (95% CI)**	***P*-value**
**Sex**				
Female	Reference	–	Reference	–
Male	3.0394 (1.4362–6.4321)	0.004	3.6455 (1.1323–11.7361)	0.030
Age	1.0071 (0.9785–1.0365)	0.630	NA	NA
Smoking index	1.0109 (1.0015–1.0203)	0.023	0.9999 (0.9872–1.0128)	0.993
BMI	0.9517 (0.8935–1.0138)	0.125	NA	NA
SGRQ scores	1.0653 (1.0443–1.0866)	<0.05	1.0395 (1.0140–1.0656)	<0.05
**GOLD stage**				
GOLD 1	Reference	–	Reference	–
GOLD 2	2.0500 (0.8845–4.7511)	0.094	1.3233 (0.4750–3.6867)	0.592
GOLD 3	8.9167 (3.8123–20.8542)	<0.05	2.6184 (0.8800–7.7903)	0.084
GOLD 4	9.3333 (3.0332–28.7195)	<0.05	1.9026 (0.4135–8.7543)	0.409
COPD years	1.1208 (1.0536–1.1924)	<0.05	1.1044 (1.0209–1.1947)	0.013
PLR	1.0093 (1.0044–1.0142)	<0.05	1.0160 (1.0051–1.0270)	0.004
SII	1.0011 (1.0004–1.0018)	0.002	1.0002 (1.0000–1.0041)	0.086
SIRI	1.5203 (1.1914–1.9399)	0.001	1.3994 (0.9070–2.1592)	0.129
PLT/MPV	0.9909 (0.9502–1.0333)	0.669	NA	NA
PLT/RDW	0.9966 (0.9335–1.0640)	0.919	NA	NA

*OR, odds ratio; CI, confidence interval; PLR, platelet–lymphocyte ratio; SII, platelet × neutrophil/lymphocyte; SIRI, monocyte × neutrophil/lymphocyte; PLT/RDW, platelet counts–red cell distribution width ratio; PLT/MPV, platelet counts–mean platelet volume ratio*.

**Table 3:** Multivariate logistic regression analysis for moderate exacerbation.

	**Severe**		**Moderate**		**Mild**		**Frequency**	
	**O R (95% CI)**	***P*-value**	**OR (95% CI)**	***P-*value**	**OR (95% CI)**	***P-*value**	**OR (95% CI)**	***P*-value**
SGRQ score	1.0630 (1.0436–1.0827)	<0.05	–	–	–	–	1.0444 (1.0272–1.0619)	<0.05
PLR	1.0093 (1.0006–1.0180)	0.035	–	–	–	–	–	–
SII	1.0019 (1.0003–1.0036)	0.021	1.0009 (1.0002–1.0016)	0.007	–	–	–	–
SIRI	1.3785 (0.9917–1.9163)	0.056	–	–	–	–	–	–
PLT/MPV	–	–	–	–	0.9544 (0.9136–0.9971)	0.037	0.9498 (0.9089-0.9925)	0.022

*OR, odds ratio; CI, confidence interval; PLR, platelet–lymphocyte ratio; SII, platelet × neutrophil/lymphocyte; SIRI, monocyte × neutrophil/lymphocyte; PLT/RDW, platelet counts–red cell distribution width ratio; PLT/MPV, platelet counts–mean platelet volume ratio*.

### Predictive Ability of Ratio Indexes

**Figure 2:**
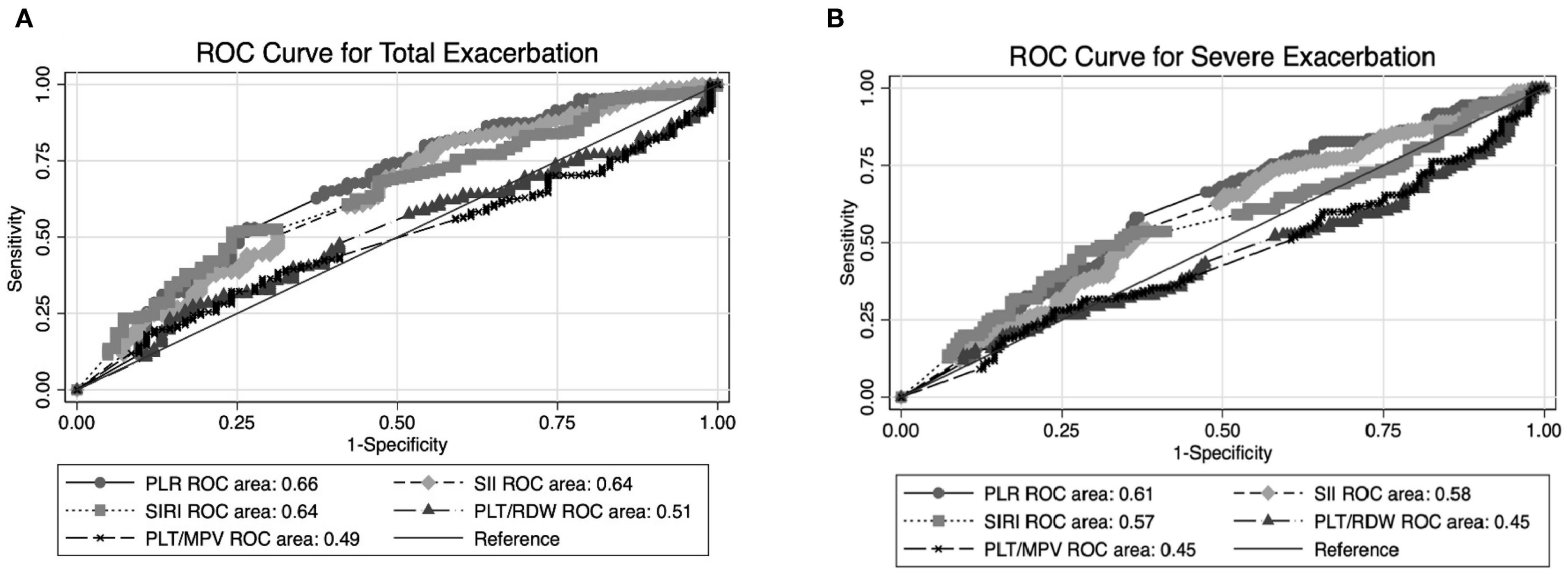
Graph shows results of ROC analysis for predicting exacerbation among stable chronic obstructive pulmonary disease (COPD) patients by different ratio indexes (PLR, SII, SIRI, PLT/MPV, and PLT/RDW). **(A)** ROC curve analysis of ratio index to predict total exacerbation. **(B)** ROC curve analysis of ratio index to predict severe exacerbation. ROC, receiver operating characteristic, PLR, platelet–lymphocyte ratio; SII, platelet × neutrophil/lymphocyte; SIRI, monocyte × neutrophil/lymphocyte; PLT/RDW, platelet counts–red cell distribution width ratio; PLT/MPV, platelet counts–mean platelet volume ratio.

To compare the predictive ability of the ratio indexes, we drew the ROC of PLR, SII, SIRI, PLT/RDW, and PLT/MPV alone to predict total and severe exacerbation events. The AUC values of each index were presented in Figure 2. In both figures, the predictive ability of PLR exceeded that of other ratio indexes individually. It appeared that SII and SIRI also behave well in predicting exacerbation events. We further compared Figures 2A,B and found that the ability of all these ratio indexes (including PLR) to predict total exacerbation is universally better than severe exacerbation.

### Predictive Ability of Combined Indexes

**Figure 3:**
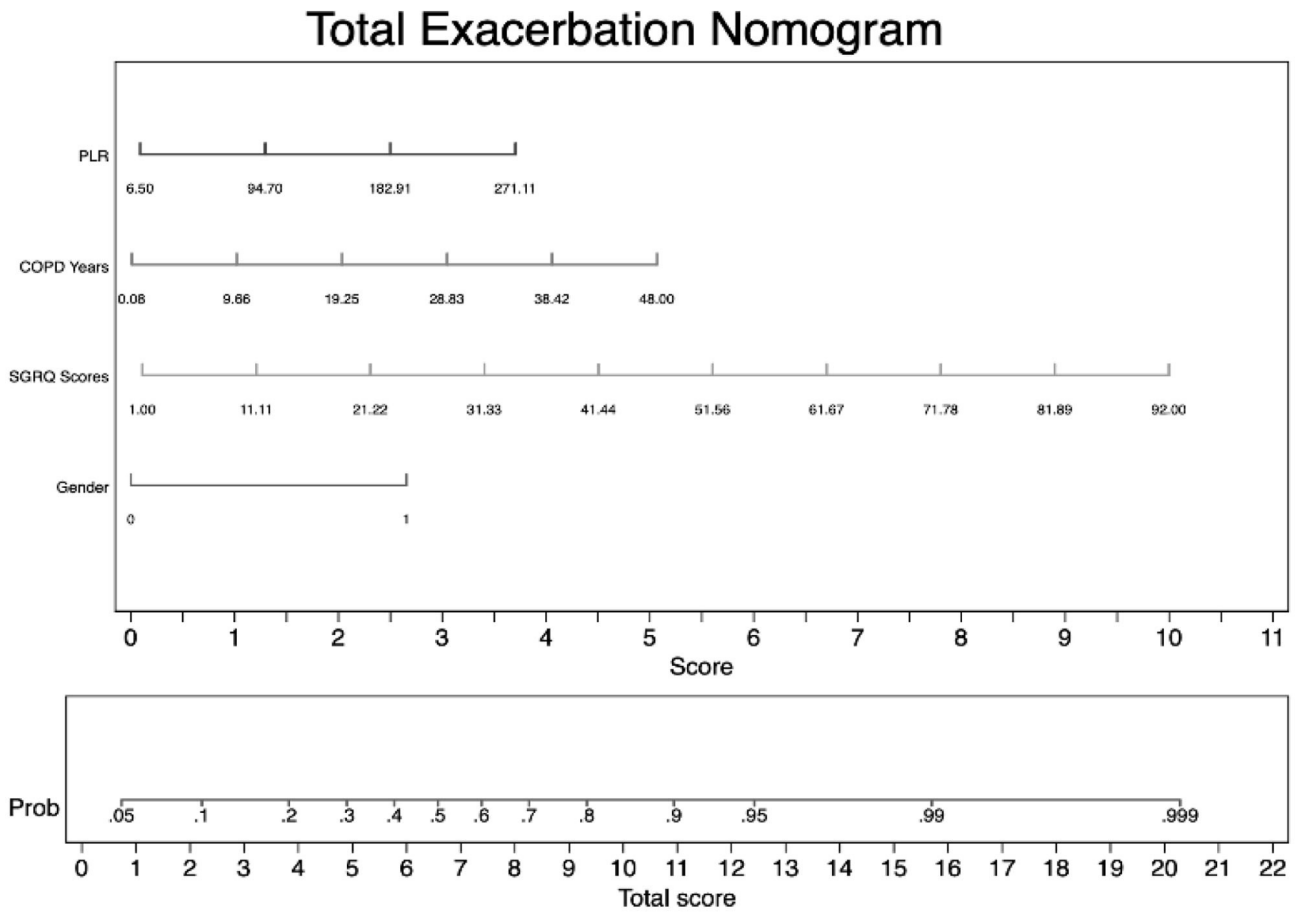
Nomogram for predicting total exacerbation in patients with stable COPD. To use the nomogram for predicting each individual patient's exacerbation risk, first we locate the range of each variable on the horizontal scale and draw a line vertically to the bottom score line to determine the corresponding points. Then, we sum up the points of all three variables and locate the total score on the total score scale. Finally, we draw a vertical line from the dot on the total sore line to the upward risk probability line to calculate the risk of exacerbation.

**Figure 4:**
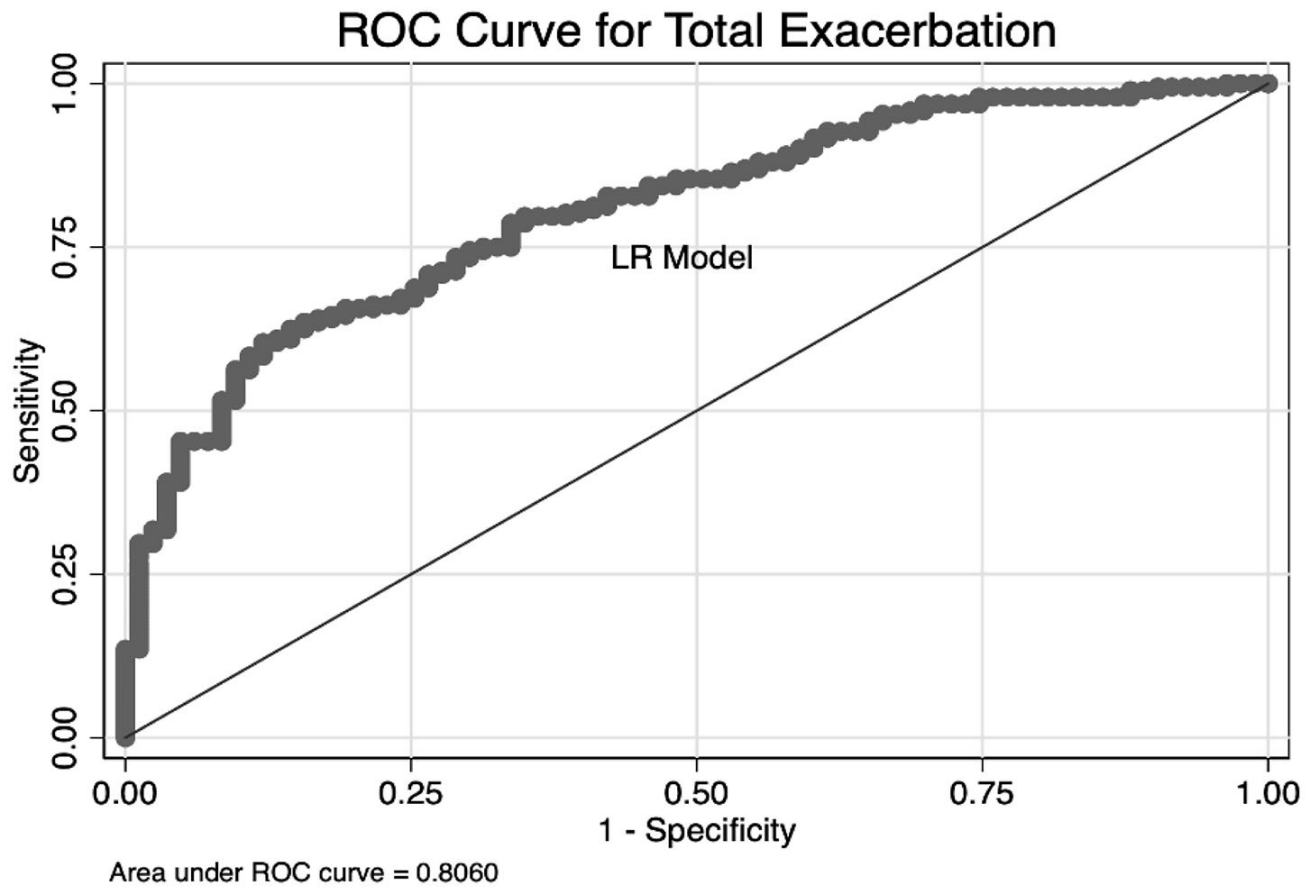
Graph shows the results of ROC curves analysis for predictive models. Predictors were arising from multivariate logistic regression of total exacerbation among stable COPD patients [sex, COPD years, St. George's Respiratory Questionnaire (SGRQ) scores, and PLR].

As we have mentioned in multivariate logistic regression, PLR was closely related to total exacerbation and severe exacerbation. Due to the superiority of PLR in predictive ability, we then investigated the predictive discrimination and accuracy of the logistic model: combining hemogram index PLR with other significant indexes (sex, COPD years, and SGRQ scores) to increase predictive sensitivity. Based on these four predictive indexes, we first established a predictive nomogram for total exacerbation in stable COPD patients. As shown in Figure 3, the result of the nomogram plot showed that PLR is one of the contributing factors. For every 88.2-unit increase in PLR value, the score in the exacerbation system will increase by one point. In addition, for every 9.58 years of increase in the course of COPD, or for every 1.11 increase in SGRQ scores, a person's exacerbation score will increase by one unit. The predictive accuracy of index combinations is exhibited in Figure 4.

**Figure 5:**
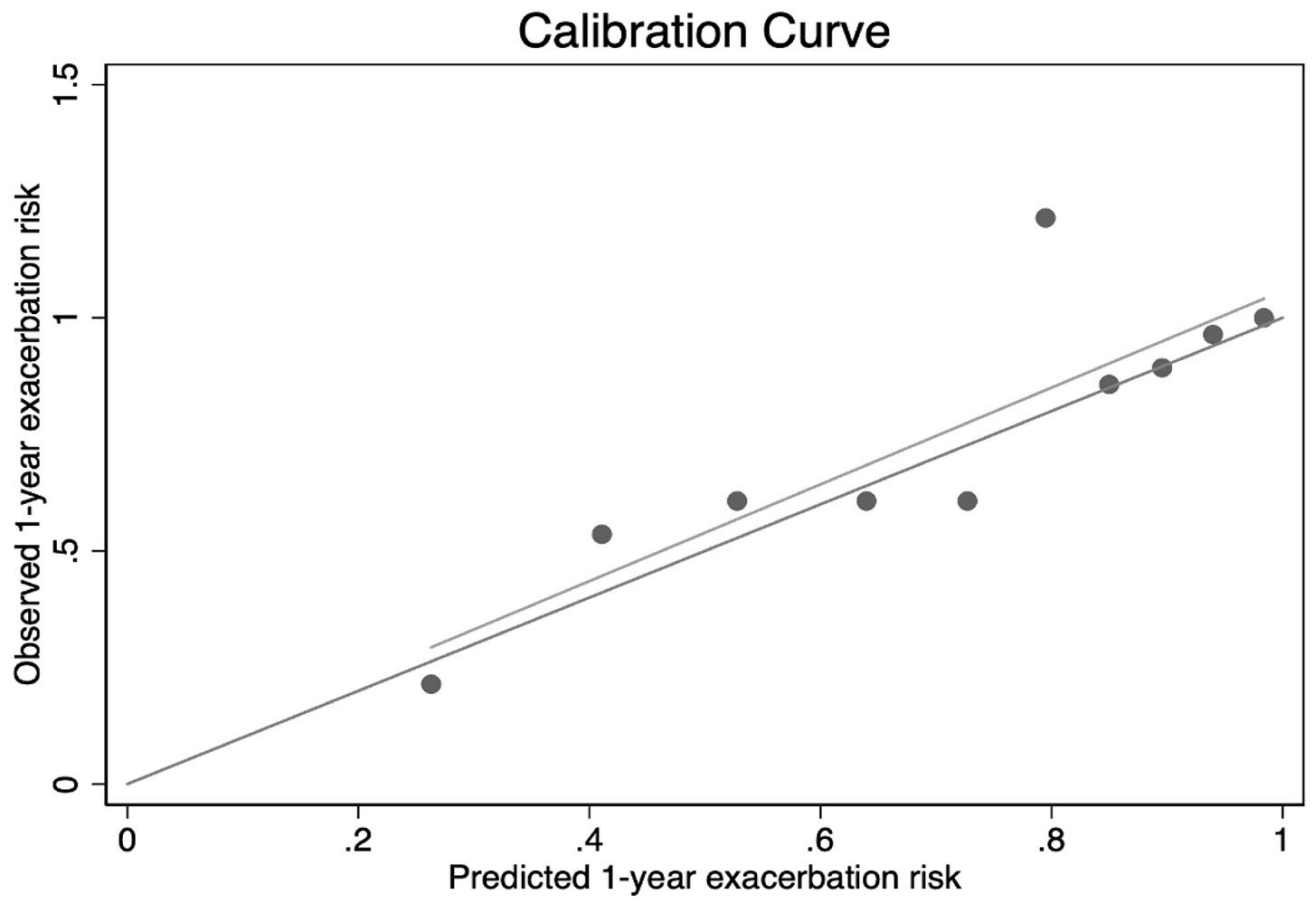
Calibration curve of combined indexes (PLR, sex, COPD years, and SGRQ scores) to predict total exacerbation of COPD patients. Result of Hosmer–Lemeshow fitness test: χ^2^ = 6.37, *P* = 0.606.

We obtained the best AUC of 0.806 when using a combination of PLR, sex, COPD years, and SGRQ scores. As we expected, the predictive capacity of PLR was significantly augmented, combining with other indexes. These results also suggested that the model showed good discriminability. Furthermore, the calibration curve of the model and Hosmer–Lemeshow fitness test are performed to assess the model's accuracy. The slope of the calibration curve was close to 1.0, which indicated that there was good consistency between predictive exacerbation risk and actual exacerbation risk (Figure 5). The result of the Hosmer–Lemeshow fitness test (χ^2^ = 6.37, *P* = 0.606) confirmed the accuracy. Therefore, the strong evidence in our study supported that a combination of PLR with other indexes is a qualified combination to predict total exacerbation. To conversely prove the superiority of PLR, we combine the three known indexes related to COPD (sex, COPD year, and SGRQ scores) to predict total exacerbation. The AUC is 0.7769, which is lower than the four indexes. The result suggested that PLR did play an important role, especially combined with other predictors (Supplement 2).

## Discussion

Exacerbation of COPD refers to the acute event during the clinical course of COPD (18), which is significantly associated with the clinical outcomes of patients with COPD by worsening clinical symptoms, declining lung function, and increasing mortality. Therefore, identifying a simple and reliable marker to predict exacerbation in stable COPD patients is becoming more and more urgent. By providing a comprehensive assessment of hemogram indexes, especially ratio indexes, this study confirmed that platelet related indexes such as PLR showed great predictive ability in an exacerbation. Also, we further explored the predictive discrimination and accuracy of index combinations. In the present study, we found that a combination of platelet-related index (PLR) reliably predicts total exacerbation of COPD patients in a stable stage.

Recently, the hemogram indexes, especially ratio indexes (PLR, SII, SIRI, PLT/RDW, PLT/MPV), have aroused wide public concern. Many pieces of research have proved that ratio indexes can be useful markers to predict bacterial infection in hospital mortality in patients with acute exacerbations of COPD (17). Studies related to PLT/RDW and PLT/MVP were not too much, but they have been verified in improving sepsis bacteremia diagnostics and even in predicting severe burn injury (14, 19). However, the present studies have not elucidated the clinical prognostic value of ratio indexes in stable COPD patients or compared the efficacy of different ratio indexes. Our study provided evidence supporting the relationship between hemogram indexes and exacerbation in the stable stage. Moreover, we aimed to contribute to growing area of research by exploring a combination of indexes, especially platelet-related indexes.

Platelet-related index, including PLR and MPV, are found to play important roles in total exacerbation of COPD (20). Praneel Kumar and Stephanie Law ascertained that PLR is significantly associated with 90-mortality in patients with acute exacerbation (21). Consistent with the literature, our study also observed that the levels of PLR are positively correlated with total and severe exacerbation events. When we refer to platelet in COPD, platelet activation is the kernel of the mechanism. Patients with COPD demonstrate increased platelet activation (16), with further activation occurring during acute exacerbations (13). In the status of hypoxemia, platelet behavior will change (22). The concrete performance is in several aspects: (1) The platelet survival time is shortened and can be reversed by oxygen treatment (23) (2) Activated platelet with lower platelet aggregate ratios standard for the increasing tendency to the aggregate formation in the circulation (22, 24) (3) Mean platelet volume is greater and in negative correlation with PaO_2_. One hypothesis was put forward to explain it. They thought the immature platelet dropping off from megakaryocytic contributed to this (23, 25, 26). (4) Activated platelet secretes platelet factor 4 and P-selectin, and platelet–monocyte aggregate is the soluble marker of platelet activation (27, 28). (5) The last formation of activation is the amount. Ashraf Faway reported that those with thrombocytosis are more likely to have worse dyspnea, functional status, and quality of life compared with those with lower platelet count (27). In addition, thrombocytosis was an independent predictor of both inpatient mortality and 1 year mortality among survivors to discharge (29). All that are discussed are the external performance of platelet activation. In summary, all the evidence suggested that the relationship between platelet-related index and exacerbation exactly existed. Although PLR only reflects the change of platelet in amount, the underlying mechanism should come back to the platelet activation. When we use the combination of PLR with other indexes, we have a more comprehensive understanding of platelet activation in COPD patients. That may be the reason why index combination can predict exacerbation in COPD better than an individual one. Our results also confirmed the association between platelet activation and COPD exacerbation in a way. Also, we found that PLT/MPV is greatly correlated with mild exacerbation (Table 3). It is probably because the index PLT/MPV itself subsumes platelet counts and mean platelet volume, which can better reflect platelet activation. Our results offer a new thought for exploring marker in mild exacerbation of COPD patients. With the in-depth study, reactive oxidative stress (ROS) is proved to be associated with abnormal inflammation and exacerbation in patients with COPD (1, 30). Furthermore, someone holds the opinion that ROS itself is one component of inflammation by activating the oxidant-sensitive transcription factors to improve the transcription of pro-inflammatory genes in COPD (31). During the procedure of ROS, oxidized low-density lipoprotein can activate transcription factor, nuclear factor kappa B, which is related to the pathogenesis of COPD. PI3K is also activated in ROS, which can exaggerate inflammation. ROS may be the upstream in the whole regulatory networks, and platelet is one of the targets. Our result (Table 2) is in accordance with theirs; the higher the PLR level is, the more possible exacerbation happens.

SIRI and SII comprehensive indexes can indicate systemic inflammation and immune status. Huaping Huang reported the prognostic value of preoperative SII in patients with cervical cancer (14). Previous research also proved the clinical utility in clear cell renal cell carcinoma and esophageal squamous cell carcinoma (32, 33). They attributed the results to the synthesis and comprehensiveness of SII and SIRI. With regard to COPD, it is widely recognized that airflow limitation is related to abnormal inflammation, which is a multicomponent inflammatory response (34). However, how SII and SIRI functioned in COPD has not been determined. SIRI is one kind of index containing platelet counts, monocytes counts, and lymphocytes counts. We confirmed that SII and SIRI are positively related to total and severe exacerbation in COPD patients in our study. What is more, the ROS may be a linking bridge between them. In ROS, oxidized low-density lipoprotein also plays a great part in other aspects. It can activate the chemotaxis of monocytes and increase the expression of monocyte chemotactic protein-1 (30). On one hand, under the influence of chemotaxis, monocyte counts will increase. On the other hand, through platelet activation, the cell surface adhesion molecules will release to recruit more monocytes (28). Someone who explored the clinical use of intercellular adhesion molecule 1 to be the biomarker of COPD found that intercellular adhesion molecule 1 is produced by bronchial epithelial cells and endothelial cell aiming to activate lung macrophages (35), maybe the discovery is also suitable in the peripheral circulation. In the present study, the predictive capacity of SIRI far exceeds PLT/MPV and PLT/RDW, whether in a total or severe exacerbation.

SII concludes platelet counts other than SIRI. It takes two aspects, inflammation and immunity, into consideration. Someone assumed that good performance of SII might owe to the integration of three indexes, which avoid being easily affected by individual differences and show greater stability (2, 21, 36). That may be exactly the same as SIRI. In our study, SII showed the same superiority as SIRI in predicting the exacerbation of COPD patients. The higher SII and SIRI generally heralds the higher systemic inflammatory level. Evidence supports that systematic inflammation is commonly seen in COPD, especially during exacerbation because of the increasing circulating cytokines, chemokines, and acute-phase proteins. However, until now, we cannot figure out whether the systematic inflammation is a “spill over” from the lung or a parallel abnormality (1). So, the extrapulmonary effects cannot be ignored (37). Further research in systemic inflammation of COPD is needed.

As a regional study, the study reflected the results of the particular population and had its limitations. First, the sample size in our study is considerably limited. Sensitivity and specificity may be improved as the samples' quantity increases. Second, compared to other western large-scale COPD clinical studies, the subjects we enrolled may be older, and we contained fewer women. So, when we extrapolated the results in population, it is not suitable for young and female COPD patients. Third, although we found that the hemogram indexes, especially PLR, are correlated with exacerbation, the mortality, and rate of descent in pulmonary function have not been evaluated.

Despite these limitations, our study still provides some new insights into the hemogram indexes and exacerbation of COPD. With the multivariate regression analysis, the result demonstrated that ratio index PLR was significantly related to the exacerbation of COPD. Also, they showed the high-powered efficacy to predict exacerbation after combining with other indexes. Furthermore, we estimated the discrimination and accuracy of combined indexes to predict exacerbation. The information will be useful in phenotyping COPD patients. Besides, it is conducive to screen high-risk patients during the stable stage. Targeted and appropriate interventions for a certain population should be performed in an early stage.

## Data Availability Statement

The raw data supporting the conclusions of this article will be made available by the authors, without undue reservation.

## Ethics Statement

The studies involving human participants were reviewed and approved by Clinical Research Ethics Committee of Huadong Hospital. The patients/participants provided their written informed consent to participate in this study.

## Author Contributions

JZ and HZ contributed to study design. XF, JH, FZ, XJ, HB, MZ, FH, YQ, ZJ, WG, BG, LY, JW, and SL contributed to participants enrollment and contributed to study management and data collection. XL and HG contributed to manuscript writing, data analyses, and manuscript revision. All authors have read and approved the final manuscript.

## Conflict of Interest

The authors declare that the research was conducted in the absence of any commercial or financial relationships that could be construed as a potential conflict of interest.
