# Supplementary material for: The Combination of Hemogram Indexes to Predict Exacerbation in Stable Chronic Obstructive Pulmonary Disease
Source: Front Med (Lausanne). 2020 Dec 9;7:572435. doi: 10.3389/fmed.2020.572435 (PMC7769039; doi:10.3389/fmed.2020.572435)

**Supplement1.** Univariate and Multivariate Poisson regression analysis for total exacerbation

|  | Univariate model |  |  | Multivariate model |  |
| --- | --- | --- | --- | --- | --- |
|  | IRR(95%CI) | *P*-value |  | IRR(95%CI) | *P*-value |
| **Gender**  **Female**  **Male**  **Age**  **Smoking Index**  **BMI**  **SGRQ scores**  **GOLD stage**  **GOLD 1**  **GOLD 2**  **GOLD 3**  **GOLD 4**  **COPD years**  **PLR**  **SII**  **SIRI**  **PLT/MPV**  **PLT/RDW** | Reference  3.0290(1.3748-2.9945)  1.0054(0.9970-1.0177)  1.0054(1.0028-1.0081)  1.0006(0.9780-1.0237)  1.0244(1.0200-1.0289)  Reference  1.6500(1.0082-2.700)  3.1066(1.9689-4.9019)  4.6026(2.8542-7.4220)  1.0299(1.0536-1.1924)  1.0038(1.0024-1.0052)  1.0005(1.0003-1.0007)  1.1091(1.0382-1.1849)  0.8179(0.7367-0.8940)  1.0759(1.0202-1.1346) | ----  <0.05  0.166  <0.05  0.961  <0.05  ----  0.046  <0.05  <0.05  <0.05  <0.05  <0.05  0.002  <0.05  0.007 | Reference  2.6035(1.5403-4.003)  NA  1.0009(0.9979-1.0040)  NA  1.0124(1.0066-1.0181)  Reference  1.1138(0.6480-1.9143)  1.7122(1.0225-2.8671)  2.3485(1.3676-4.0330)  1.0196(1.0071-1.0321)  1.0011(1.0042-1.0843)  1.0007(1.0002-1.0012)  1.1856(0.7243-0.9157)  0.8763(0.7843-0.9614)  1.0954(1.0193-1.17772) | | ----  <0.05  NA  0.543  NA  <0.05  ----  0.697  0.041  0.002  0.002  0.474  0.010  0.001  0.004  0.013 |

**Supplement 2.** Graph shows the results of ROC curves analysis for total exacerbation among stable patients. Predictors concluded gender, COPD years, SGRQ scores.


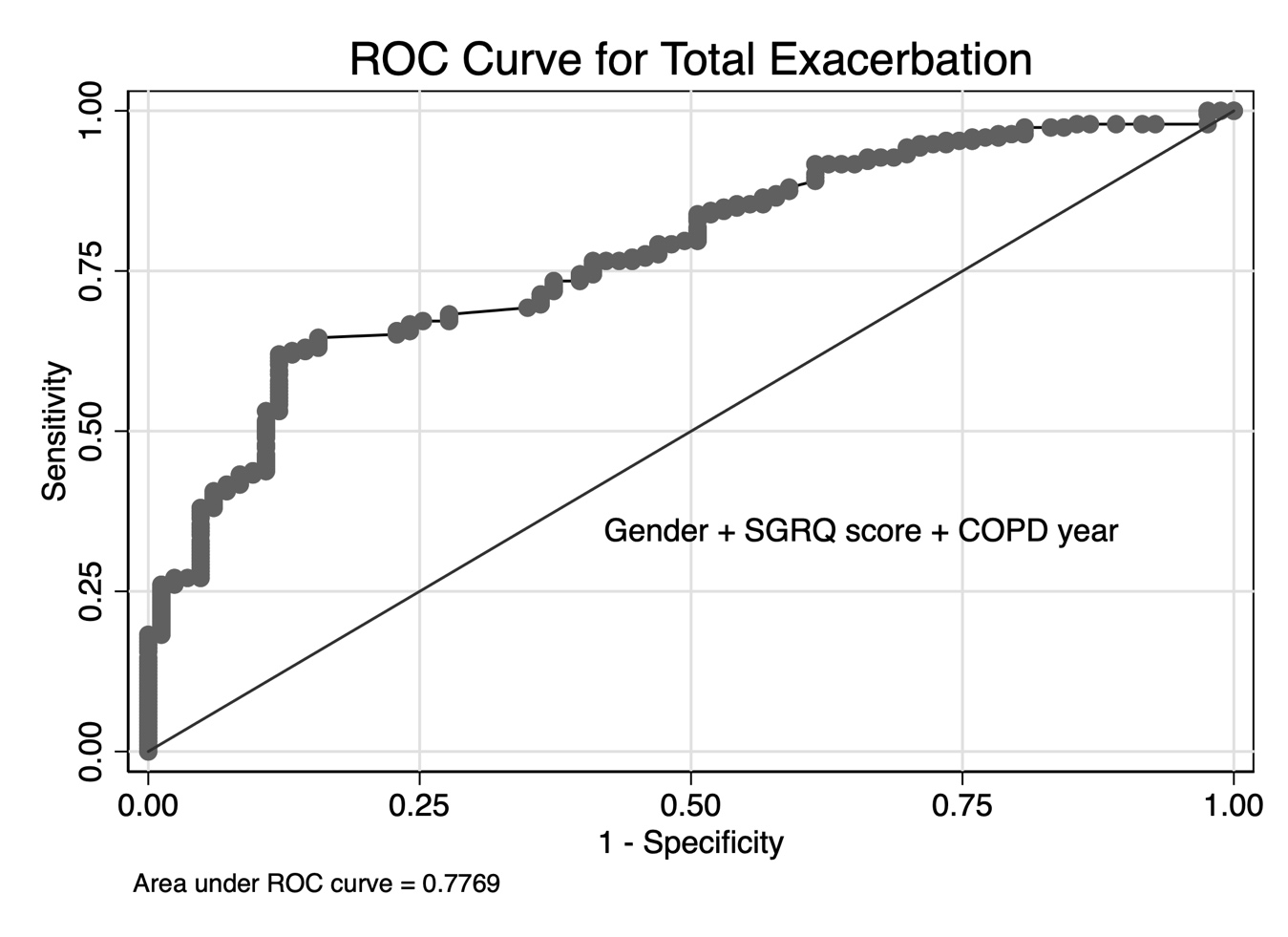

Supplement: Supplementary file 1 [file Data_Sheet_1.docx]
